# The Effect of Mirtazapine on Cisplatin-Induced Oxidative Damage and Infertility in Rat Ovaries

**DOI:** 10.1155/2013/327240

**Published:** 2013-04-22

**Authors:** Durdu Altuner, Mine Gulaboglu, Omer Erkan Yapca, Nihal Cetin

**Affiliations:** ^1^Department of Pharmacology, Faculty of Medicine, Recep Tayyip Erdogan University, 53100 Rize, Turkey; ^2^Department of Biochemistry, Faculty of Pharmacy, Ataturk University, 25240 Erzurum, Turkey; ^3^Obstetrics and Jinecology Clinic, Sorgun State Hospital, 66700 Yozgat, Turkey; ^4^Department of Pharmacology, Faculty of Medicine, Ataturk University, 25240 Erzurum, Turkey

## Abstract

Cisplatin causes infertility due to ovarian toxicity. The toxicity mechanism is unknown, but evidence suggests oxidative stress. In this study, the effect of mirtazapine on cisplatin-induced infertility and oxidative stress in rats was investigated. 64 female rats were divided into 4 groups of 16. Except for the controls that received physiologic saline only, all were administered with cisplatin (5 mg/kg i.p.) and mirtazapine (15 mg/kg p.o.) or mirtazapine (30 mg/kg p.o.) for 10 days. After this period, six rats from each group were randomly selected, and malondialdehyde (MDA), myeloperoxidase (MPO), nitric oxide (NO), total gluthatione (tGSH), gluthatione peroxidase (GPx), superoxide dismutase (SOD), and 8-hydroxy-2 deoxyguanine (8-OH Gua) levels were measured in their ovarian tissues. Reproductive functions of the remaining rats were examined for 6 months. The MDA, MPO, NO groups and 8-OH Gua levels were higher in the cisplatin-treated groups than the controls, which was not observed in the mirtazapine and cisplatin groups. GSH, GPx, and SOD levels were reduced by cisplatin, which was prevented by mirtazapine. Cisplatin caused infertility by 70%. The infertility rates were, respectively, 40% and 10% for the 15 and 30 mg/kg mirtazapine administered groups. In conclusion, oxidative stress induced by cisplatin in the rat ovary tissue causes infertility in the female rats. Mirtazapine reverses this in a dose-dependent manner.

## 1. Introduction

Cisplatin, a platinum derivative, is a chemotherapeutic agent used for the treatment of solid tumors [1]. Cisplatin is asserted as an intermediately gonadotoxic agent [2]. Moreover, it has been demonstrated that cisplatin-associated infertility is caused by the toxic effect on the primordial follicles. Since the primordial follicles are not able to regenerate, the damage caused by the exposure to toxic agents may lead to ovarian insufficiency and infertility [3]. The severe adverse effects occurring during cancer chemotherapy restrict the appropriate use of anticancer drugs [4]. The anti-cancer drugs, particularly those used in early childhood and in reproductive period, may cause several complications such as ovarian insufficiency and infertility [5]. Therefore, in recent years, trials have been initiated on several methods to prevent infertility in patients given chemotherapy [6]. The mechanism of action of cisplatin toxicity on ovaries has not been explained thoroughly. However, it is thought that increased production of free oxygen radicals and decreased production of antioxidants have an impact on the occurrence of cisplatin toxicity [7, 8]. It has been claimed that organ damage related to free radicals occurs as a consequence of disrupted antioxidant defense mechanisms. Moreover, it was reported that the toxicity caused by cisplatin in the tissues was closely related to the increased lipid peroxidation [9, 10]. This literature knowledge suggests that antioxidant treatment might be helpful to prevent cisplatin-related ovarian toxicity and therefore infertility due to this toxicity. Mirtazapine that we tested in this trial is an antidepressant drug used in the treatment of major depression. In recent years, researchers have begun to conduct studies about the antioxidant activity of mirtazapine along with its antidepressant effect. It was reported that it could be used as a cell protector because of its inhibitor effects particularly on the antioxidant parameters [11, 12]. In literature research, we found no information about preventing infertility with mirtazapine in rats with oxidative ovarian damage due to cisplatin administration. Thus, the purpose of this study was to demonstrate whether mirtazapine would be efficacious for preventing infertility occurred in rats with oxidative ovarian damage due to cisplatin administration, and to define the association of oxidative stress in ovarian tissues with infertility.

## 2. Materials and Method

### 2.1. Animals

For the experiment, a total of 96 albino Wistar female rats weighing between 165-170 g were used. The rats were provided by the Medical Experimental Practice and Research Center of Ataturk University. The animals were kept in room temperature (22°C) in groups and were fed ad libitum. Ataturk University Local Ethical Committee of Experimental Animals approved that all the steps of this study were compliant with ethical rules.

### 2.2. Chemical Substances

The thiopental sodium used in the experiment was provided by Ibrahim Etem Ulagay (Istanbul, Turkey); cisplatin and mirtazapine were provided by Organon Pharmaceuticals (New Jersey, USA).

The rats used in this study were divided into four groups—cisplatin group (Cis), 15 mg/kg of mirtazapine + cisplatin group (Mirt-15), 30 mg/kg of mirtazapine + cisplatin group (Mirt-30), and control group (C). The rats in Cis group (*n* = 16) were injected with cisplatin at a dosage of 5 mg/kg intraperitoneally (i.p.). The rats in Mirt-15 group (*n* = 16) and those in Mirt-30 group (*n* = 16) were given mirtazapine orally at dosages of 15 mg/kg and 30 mg/kg, respectively. Subsequently, one hour later, cisplatin was administered intraperitoneally 5 mg/kg. The rats in group C (*n* = 16) were given distilled water orally in equal volumes. This procedure was applied throughout 10 days. At the end of the period, six rats from each group were randomly selected and sacrificed using high-dose anesthetic (50 mg/kg of thiopental i.p.). Their ovaries were removed, and the levels of malondialdehyde (MDA), myeloperoxidase (MPO), nitric oxide (NO), total gluthatione (tGSH), gluthatione peroxidase (GPx), superoxide dismutase (SOD), and 8-hydroxy-2 deoxyguanine (8-OH Gua), were measured.

Other animals were housed in an appropriate environment for reproduction. Reproductive functions of the remaining rats (*n* = 10 of each group) were examined for 6 months. Rats not getting pregnant and not giving birth during this period were accepted as infertile.

### 2.3. Biochemical Analysis of Ovarian Tissue

Whole ovarian tissue was weighed and homogenized on ice with 2-mL relevant buffer. The buffers were 0.5% hexadecyltrimethyl ammonium bromide (pH 6), potassiumphosphate buffer formyeloperoxidase analysis, 1.15% potassium chloride solution for malondialdehyde analysis, and pH 7.5 phosphate buffer for the other analyses. Then, they were centrifuged at 4°C, 10,000 rpm for 15 min. The supernatant was used for analysis.

### 2.4. MDA Analysis

The concentrations of ovarian mucosal lipid peroxidation were determined by estimating MDA using the thiobarbituric acid test [13]. 0.5 mL homogenate was added to a solution containing 0.2 mL of 80 g/L sodium lauryl sulfate, 1.5 mL of 200 g/L acetic acid, 1.5 mL of 8 g/L of 2-thiobarbiturate, and 0.3 mL of distilled water. The mixture was incubated at 98°C for 1 h. Upon cooling, 5 mL of n-butanol : pyridine (15 : l) was added. The mixture was vortexed for 1 min and centrifuged for 30 min at 4000 rpm. The absorbance of the supernatant was measured at 532 nm. The standard curve was obtained by using 1,1,3,3-tetramethoxypropane.

### 2.5. MPO Analysis

The activity of MPO in the total homogenate was measured according to the method of Wei and Frenkel with some modifications [14]. The supernatant was used to determine MPO activity using 1,3 mL 4-aminoantipyrine-2% phenol (25 mM) solution. 25 mM 4-aminoantipyrine-2% phenol solution and 0.0005% 1.5 mL H_2_O_2_ were added and equilibrated for 3-4 min. After establishing the basal rate, a 0.2 mL sample suspension was added and quickly mixed. Increases in absorbance at 510 nm for 4 min at 0.1 min intervals were recorded.

### 2.6. NO Analysis

Nitric oxide levels were measured by the Griess reaction [15, 16]. Nitric oxide measurement is difficult because of its brief half life. Therefore, nitrate and nitrite levels, which are stable end products of nitric oxide metabolism, were used. 100 *μ*L Griess reagent and 100 *μ*L of metaphosphoric acid were added to the supernatant, and a deep purple azo compound occurred. The Griess reagent consists of 0.5 g sulfanilamide, 12.5 g phosphoric acid, and 0.05 g N-(1-napthyl)-ethylenediamine in 500 mL distilled water. Absorbance of the deep purple azo compound was measured at 540 nm wave length by photometric measurement. This azo chromophore accurately determines nitrite concentration as a marker of NO.

### 2.7. tGSH Analysis

The amount of GSH in the total homogenate was measured according to the method of Sedlak and Lindsay with some modifications [17]. 1500 *μ*L of measurement buffer (200 mM Tris-HCl buffer containing 0.2 mM EDTA at pH 7.5), 500 *μ*L of supernatant, 100 *μ*L of 5,5-dithiobis (2-nitrobenzoic acid) (10 mM), and 7900 *μ*L of methanol were added to a tube and vortexed and incubated for 30 min in 37°C. The absorbance was measured at 412 nm using a spectrophotometer. The standard curve was obtained by using reduced glutathione.

### 2.8. GPx Analysis

GPx activity was determined according to the method of Lawrence and Burk [18]. The absorbance at 340 nm was recorded for 5 minutes.

### 2.9. SOD Analysis

SOD activity was determined according to the method of Sun et al. [19]. 2450 *µ*L of measurement mixture (0.3 mM xanthine, 0.6 mM EDTA, 150 *μ*M nitroblue tetrazolium (NBT), 0.4 M Na_2_CO_3_, and 1 g/L bovine serum albumin), 500 *μ*L supernatant, and 50 *μ*L xanthine oxidase (167 U/L) was vortexed. Then it was incubated for 10 min. At the end of the reaction, formazan occurs. Absorbance of the purple-colored formazan was measured at 560 nm. 

### 2.10. Measurement of 8-OH Gua by High-Performance Liquid Chromatography (HPLC)

At first, the DNA was isolated from ovarian tissue for the measurement of 8-OH Gua, using the modified method of Shigenaga et al. [20]. Approximately, 50 mg of DNA was hydrolyzed with 0.5 mL of formic acid (60%, v/v) for 45 min at 150°C [21]. Formic acid was removed by freeze-drying. Before analysis by HPLC, DNA samples were redissolved in the eluent (final volume, 200 *μ*L).

The amount of 8-OH Gua and guanine (Gua) was measured by using an HPLC system equipped with an electrochemical detector (HP Agilent 1100 module series, E.C.D. HP 1049 A), as described previously [21, 22]. The amount of 8-OH Gua and Gua was analyzed on a 250 mm × 4.6 mm Supelco LC-18-S reverse-phase column. The mobile phase was 50 mM potassium phosphate, pH 5.5, with acetonitrile (97-volume acetonitrile and 3-volume potassium phosphate), and the flow rate was 1.0 mL/min. The detector potential was set at −0.80 V for measuring the oxidized base. Gua and 8-OH Gua (25 pmol) were used as standards. The 8-OH Gua levels were expressed as the number of 8-OH Gua molecules/10^5^ Gua molecules [23].

### 2.11. Statistical Analysis

All data were subjected to one-way analysis of variance (ANOVA) using statistical package for the social sciences (SPSS) version 15.0 software. The differences among groups were analyzed using the least significant difference (LSD) option and a *P* value < 0.05 was considered to be statistically significant. The results are presented as the mean ± standard error of the mean (SEM).

## 3. Results

**Figure 1:**
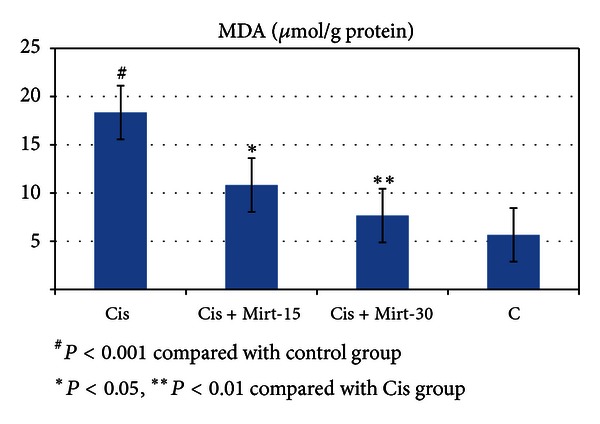
MDA levels of study groups.

**Figure 2:**
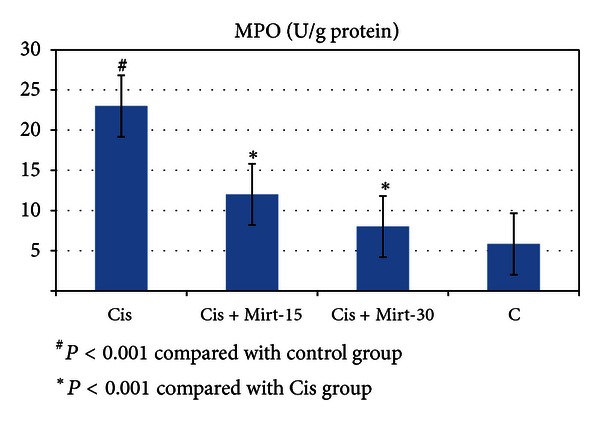
MPO activity of study groups.

**Figure 3:**
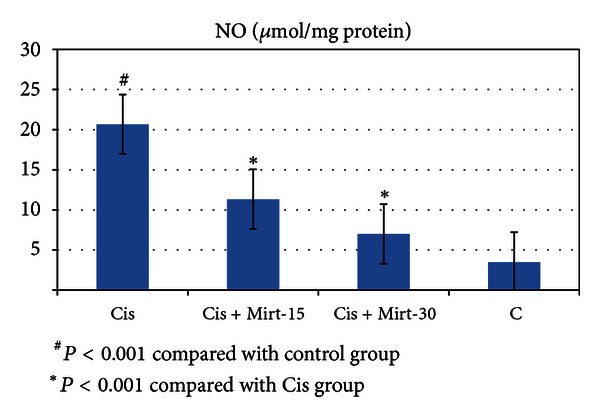
NO levels of study groups.

**Figure 4:**
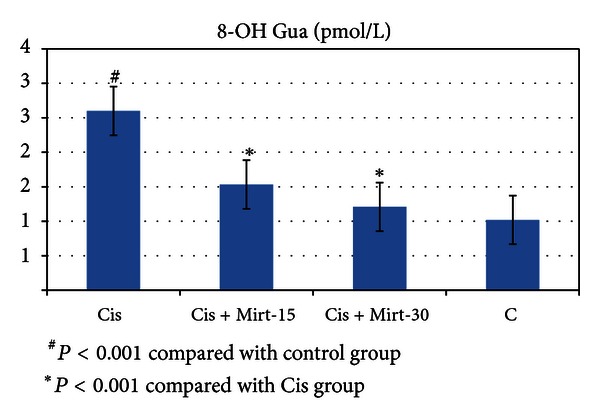
8-OH Gua levels of study groups.

**Figure 5:**
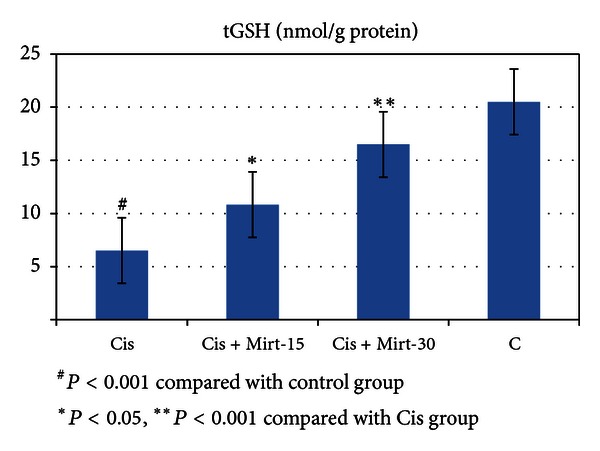
tGSH levels of study groups.

**Figure 6:**
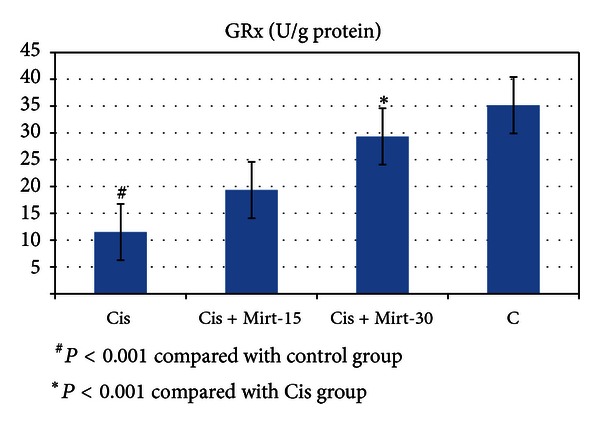
MPO activity of study groups.

**Figure 7:**
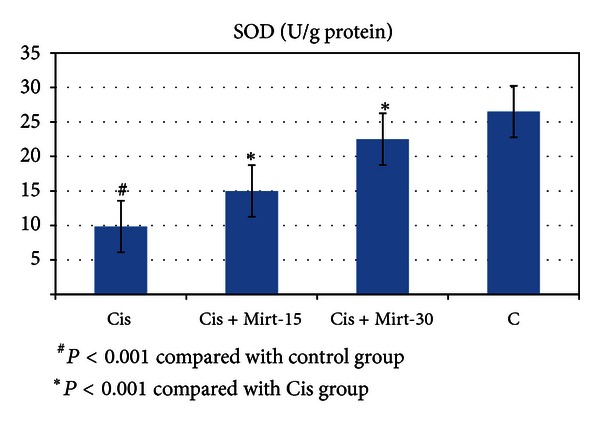
SOD activity of study groups.

All oxidant and antioxidant parameters, measured in the ovarian tissue of the rat groups, are shown in Table 1. The amounts of MDA found in the ovarian tissue of the Cis group rats were higher than the C group (*P* < 0.001). The MDA levels of the Mirt-15 and the Mirt-30 groups were found to be less than the Cis group (resp., *P* < 0.05, *P* < 0.01) (Figure 1). MPO, NO, and 8-OH Gua levels in the ovarian tissues of the rats in the Cis group were found to be the highest (*P* < 0.001 compared with the C group). MPO, NO, and 8-OH Gua levels of the Mirt-15 and the Mirt-30 groups were found to be lower than the Cis group (*P* < 0.001) (Figures 2, 3, and 4). The Cis group had the lowest amount of tGSH, one of the nonenzymatic antioxidant parameters, in the groups (*P* < 0.001 compared with the C group). The amount of tGSH in the Mirt-15 and the Mirt-30 groups was found to be higher than the Cis group (resp., *P* < 0.05, *P* < 0.001) (Figure 5). GRx activities, which is an enzymatic antioxidant parameter, measured in the Cis group were lower than in the C group (*P* < 0.001). GRx activity of the Mirt-30 group was higher than in the Cis group (*P* < 0.001) (Figure 6). The activities of SOD, another enzymatic antioxidant parameter, were measured lower than those in the C group (*P* < 0.001). The activities of SOD in the Mirt-15 and the Mirt-30 groups were measured higher than in the Cis group (*P* < 0.001) (Figure 7).

As shown in Table 2, Cisplatin caused infertility by 70% in the Cis group. The infertility rates decreased by 40% and 10% for the Mirt-15 and Mirt-30 groups, respectively.

## 4. Discussion

In this study, it was researched whether mirtazapine was efficacious to prevent infertility occurring in rats with cisplatin-induced oxidative ovarian damage. The data obtained in the study demonstrated that cisplatin caused significant oxidative stress in the ovarian tissues of rats. Moreover, in the Cis group, where oxidative stress was significant, the rate of infertility was significantly higher in comparison with the other groups. Cisplatin is used in the treatment of many solid tumors, mainly testicular and ovarian tumors. However, it was reported that cisplatin caused severe adverse effects such as nephrotoxicity, neurotoxicity, gastric toxicity, and infertility [24]. It was reported that chemotherapeutic medicines leading to either temporary or permanent infertility severely affected the ovaries and hormonal balance [25]. The deterioration of the oxidants/antioxidants balance in the tissues in favour of the oxidants is considered as oxidative stress. As the results of our study demonstrated, in ovarian tissues of animals administered cisplatin, there was an increase in the levels of MDA and MPO, which are oxidant parameters, while the levels of antioxidants such as tGSH, GPx, and SOD were decreased. MDA is the end-product of lipid peroxidation. Lipid peroxidation is known to be the most harmful effect of free radicals in the cell [26]. It was reported that cisplatin caused oxidative damage in the ovarian tissue increasing MDA concentration and decreasing GSH concentration [27]. Furthermore, in oxidative ovarian damage occurring due to ischemia reperfusion, both MDA and MPO levels were found to be significantly increased in comparison with the healthy ovarian tissue [28]. In our study, for ovarian tissues of the Cis group rats in which MDA was found to be increased, MPO activity was also increased. MPO, which is known to play important roles in tissue damage, is reduced to hypochlorous acid in the presence of hydrogen peroxide and chloride anions. The hypochlorous acid is a powerful oxidant and causes damage to vascular endothelium [29]. There was no previous study about that effect of mirtazapine over MPO activity in ovarian tissue. However, in the kidney tissue of rats given cisplatin, a significant increase in MPO activity was noticed [30]. This knowledge in the literature supports the results of our study. In the ovaries of the rats given cisplatin, GSH and GPx levels were decreased in comparison with the C group. GSH is a nonenzymatic endogenous antioxidant parameter, but GPx is an enzymatic endogenous antioxidant. Under physiological conditions, the oxidant/antioxidant balance is maintained with predominance of antioxidants. The disruption of this equilibrium causes tissue damage named oxidative stress. Therefore, oxidant/antioxidant balance is used to assess if tissue damage emerges [31]. GSH, an endogenous antioxidant, protects the cells against oxidative damage, keeping the –SH groups of proteins reduced and preventing them from reacting with free radicals [32]. GPx reduces oxidized glutathione (GSSG) by transferring one electron from NADPH to the disulfide bonds of GSSG [33]. SOD, another protective enzyme against free oxygen radicals, catalyzes the transformation of superoxide molecule into hydrogen peroxide and molecular oxygen [34]. Low SOD activity found in ovarian tissues of rats given cisplatin indicates that the oxidative stress has occurred. In ovarian tissues of rats given cisplatin, the concentration of 8-OH Gua was found to be significantly higher in comparison with Mirt-15, Mirt-30, and C groups. The 8-OH Gua is an important marker reflecting DNA oxidation and is formed by breaking off the hydrogen from the nucleic acids with the action of toxic oxygen radicals such as hydroxyl radical in injured tissues [35]. Cisplatin creates inchain and interchain cross-linking by interacting with DNA. This cross-link formation inhibits the transcription and replication of DNA. If cisplatin-induced DNA is not repaired, cell death occurs [36]. There are studies demonstrating that 8-OH Gua concentrations significantly increase in damaged ovarian tissue caused by oxidative stress in comparison with healthy tissues [37]. In our study, mirtazapine, which we tested to prevent cisplatin-induced oxidative ovarian damage, dose dependently decreased the concentrations of oxidant parameters and raised the concentrations of antioxidant parameters. Previous studies also reported that mirtazapine prevented cisplatin-induced oxidative damage in the kidney tissue [30].

Furthermore, mirtazapine significantly prevented cisplatin-associated infertility. In several studies conducted, it was found that the incidence of depression was high among cancer patients because of the adverse effects of chemotherapy [38]. The severity of depression and anxiety was found to be higher among infertile women in comparison with HIV-positive women and women with cancer and cardiac disorders. In some studies, it was found that the infertility was associated with anxiety disorder [39–41]. Recent studies demonstrated antioxidant and antiulcer activities of mirtazapine along with its antidepressant and sedative properties [42]. In conclusion, cisplatin leads to oxidative stress in the ovarian tissue of rats. Cisplatin also causes infertility in female rats. Mirtazapine prevented dose-dependent cisplatin-induced oxidative stress in the ovarian tissue and the infertility. It is hypothesized that mirtazapine prevents the infertility through its antioxidant, sedative, and antidepressant activities. The results of our study demonstrate that mirtazapine can be useful for preventing cisplatin-induced infertility.

## Figures and Tables

**Table 1:** Oxidative stress and DNA damage parameters in the study groups.

Groups	MDA	MPO	tGSH	GRx	SOD	NO	8-OH Gua
Cis	18.33 ± 3.04	23.17 ± 1.49	6.50 ± 0.76	9.83 ± 1.14	11.50 ± 0.76	20.67 ± 1.99	2.60 ± 0.16
Mirt-15	10.83 ± 1.62	12.17 ± 1.2	10.83 ± 0.95	15.00 ± 2.08	19.33 ± 0.88	11.33 ± 1.16	1.53 ± 0.14
Mirt-30	7.67 ± 0.88	8.00 ± 0.97	16.50 ± 1.06	22.50 ± 1.38	29.33 ± 1.54	7.00 ± 0.97	1.21 ± 0.11
C	5.67 ± 0.88	5.83 ± 0.60	20.50 ± 0.76	26.50 ± 1.18	35.17 ± 1.18	3.50 ± 0.67	1.02 ± 0.07

**Table 2:** Reproduction and infertility rate in the study groups.

Groups	Animals taken for reproduction	Animals giving birth		Infertile animals	
	*n*	*n*	%	*n*	%
Cis	10	3	30	7	70
Mirt-15	10	6	60	4	60
Mirt-30	10	9	90	1	10
C	10	10	100	0	0
